# An attempt to replicate action-effect learning and its generalization without stimulus-stimulus learning

**DOI:** 10.1007/s00426-026-02268-8

**Published:** 2026-05-04

**Authors:** Alina Ahrens, Heinrich R. Liesefeld, Jule Kinner, Markus Janczyk

**Affiliations:** Psychological Research Methods and Cognitive Psychology, Department of Psychology, University of Bremen, Hochschulring 18, D-28359 Bremen, Germany

**Keywords:** Ideomotor theory, Action-effects, Learning, Replication

## Abstract

In everyday life, we often generalize previously learned associations between our actions and their outcomes to novel, but similar situations. The present study aimed to conceptually and directly replicate results reported by Esser et al. (*Psychological Research, 87*(7), 2249–2258 2023), who reported that action–effect (A–E) learning generalizes to categorically similar stimuli. Yet, the overall picture in the published literature is not overwhelming with regard to such generalization, and hence a replication seems to be of particular importance. We present three experiments. In our conceptual replications (Experiment 1 and 2), we did not find evidence for A–E compatibility effects for either learned or similar stimuli. In the direct replication (Experiment 3), we observed evidence for successful A–E learning, but still failed to replicate generalization to similar stimuli. A critical difference between Experiments 1 and 2 and Experiment 3, however, is that stimulus-stimulus (S-S) learning was not possible in the former two experiments, but in the latter one (as was already acknowledged by Esser et al.). In sum, our results (1) suggest a critical role of S-S learning in this particular design (2) and question whether A–E learning truly generalizes to similar stimuli.

## Introduction

Imagine your first day in a new office. To begin that day feeling energized, you decide to start it with a cup of coffee. You approach the coffee machine and press the single available button, just as you would with your machine at home. Even though you are unsure whether it will make a strong espresso, a mild latte, or just a basic black (but still tasty) coffee, you anticipate that it will result in some form of coffee that helps you feel more awake. In this case, you are generalizing prior experience where pressing the same button has led to similar effects: obtaining coffee. Such generalization is often supported by various factors in everyday life. You may have learned an abstract understanding that pressing the button leads to “a coffee beverage,” rather than predicting a specific type each time. You might have used similar machines before, or perhaps you are just relying on the expectation that the result will serve your goal.

In cognitive psychology, similar phenomena are studied as generalization of action-effect (A–E) associations. The core question is whether people can (and do) apply previously learned A-E relationships to novel or varied effects, even in the absence of additional instructional or contextual support. Put simply, would the thoughts ‘I want a cappuccino’ and ‘I want a black coffee’ both lead you to the same bodily movement, namely pressing the power button on the coffee machine? As we investigate such phenomena in the context of the ideomotor principle, we will introduce this idea in the subsequent section.

### Ideomotor principle and A-E learning

The ideomotor principle addresses how actions are selected. It originates from 19^th^-century philosophical writings (e.g., Carpenter, 1852; Harleß, 1861; Herbart, 1825; James, 1890), but was largely dismissed from psychological research in the first half of the 20^th^ century. After Greenwald (1970) re-introduced the basic idea to the psychological literature, it was integrated into larger perception-action frameworks such as the Theory of Event Coding (Badets et al., 2016; Hommel et al., 2001; for reviews, see Janczyk et al., 2023; Shin et al., 2010). This sparked renewed interest in this topic within psychological research and resulted in a large amount of empirical research.

The fundamental idea of the ideomotor principle is that actions are selected and initiated by anticipating their outcomes. Doing so requires two steps (see, e.g., Elsner & Hommel, 2001): First, an agent learns the association between a bodily movement and its contingent effect, often initially by chance. This will henceforth be referred to as *action-effect (A-E) learning* and is the topic of the present study. Second, once these associations are established, a movement can later be addressed by anticipating the associated effect. This is because thinking about the effect, that is, reactivating its representation, can directly activate the corresponding bodily movement as a result of the previously learned association.

A–E learning is typically investigated in experiments consisting of two phases: In a *learning phase*, participants learn the association between an action and its effect, although the effect is typically irrelevant to the task at this stage. In a subsequent *test phase*, the influence of this association on participants’ behavior is then assessed (e.g., Eichfelder et al., 2023; Hommel et al., 2003; Janczyk et al., 2024; Pfister et al., 2011; Watson et al., 2015). Both phases can involve either a free-choice task (Berlyne, 1957), where participants freely choose their response (e.g., Elsner & Hommel, 2001; Eichfelder et al., 2023; Janczyk et al., 2024), or a forced-choice task (e.g., Elsner & Hommel, 2001; Hommel et al., 2003; Wolfensteller & Ruge, 2011), where an imperative stimulus indicates which response should be given.

In a free-choice test phase, participants are presented with one of the former effects (from the learning phase) as a stimulus and are asked to choose a response freely. The dependent variable is the proportion of responses that are congruent with the previously learned association, that is, the proportion of trials in which the participant pressed the key associated with the stimulus during the learning phase (see Elsner & Hommel, 2001, Exp. 2–4). According to ideomotor theory, congruent responses should be selected more often. If the test phase is forced-choice, a previous effect also occurs as a stimulus, but participants are instructed to respond in a particular way, either congruent or not with the learned association (see Elsner & Hommel, 2001, Exp. 1). In this case, the dependent variable is typically mean response time (RT) and percentage error (PE). According to ideomotor theory, the former effect activates the associated response through the previously established association, thereby facilitating congruent responses, slowing down incongruent ones, and increasing error rates in incongruent trials.

### Generalization and abstraction in A-E learning

If A-E learning underlies "real" behavior as illustrated in our introductory example, it should generalize to other stimuli. After all, life would be difficult if we had to re-learn from scratch using a coffee machine each time we encounter a new exemplar. Surprisingly, evidence for generalization of A–E learning to similar stimuli or higher-level categories is mixed at best. While some studies reported evidence for generalization, others failed to do so.

The first systematic study on this topic was a series of three experiments, carried out by Hommel et al. (2003). Each experiment targeted a different type of generalization. For example, Experiment 1 examined generalization from an exemplar to a superordinate category (e.g., from the exemplar “dog” to its category “animal”). First, participants completed 200 free-choice learning trials in which pressing a left or a right button led to the presentation of an effect word. For the control group, the effect words were the category words “furniture” and “animal” (originally in Spanish), while for the exemplar group, the effects were exemplars of these categories, namely “chair” and “dog”. In the subsequent forced-choice test phase, the stimuli were always the category words. Half of the participants of the control and the exemplar group were instructed to respond according to the previously learned association, while this assignment was reversed for the other half of participants. RTs were shorter with the former than with the latter assignment and this difference was the same for the control and the exemplar group. This result suggests that learning A-E associations on the exemplar level can indeed generalize to their categories. Experiments 2 and 3 reported generalization from one exemplar to another exemplar of the same category (e.g., from “dog” to “cat”) and from objects to other objects with comparable visual features (e.g., from an orange to a circle), respectively.

However, two recent studies were not successful in reporting evidence for (hierarchical) generalization (Eichfelder et al., 2023; Janczyk et al., 2024). Eichfelder et al. (2023) employed a free-choice task in the learning and the test phase. Even though they used stimuli and effects similar to Experiment 1 of Hommel et al. (2003) they found that participants selected responses congruent with the learned association only when the prime stimulus exactly matched the original effect (i.e., in the control group). In contrast, exemplars from the same category did not produce the same response congruency bias, indicating that no generalization occurred. Janczyk et al. (2024) used a similar experimental design and found evidence for generalization only in their Experiment 2. However, this experiment did not investigate hierarchical generalization between a category and its exemplars, but from a visual depiction of a word to its written counterpart (e.g., a picture of a cat and the word “cat”). According to the authors, this putative generalization effect might be attributed to phonological recoding, when participants internally vocalize the corresponding word when confronted with a visual stimulus. Hence, they concluded that if A-E learning generalizes, it only does so under very specific circumstances.

In summary, neither of these two studies provided evidence for (hierarchical) generalization effects. One important difference between the studies of Eichfelder et al. (2023) and Janczyk et al. (2024) and that of Hommel et al. (2003) is that the former studies employed free-choice test phases. Several recent studies, however, reported that the distribution of the individual choice-congruency effects in free-choice tasks is bimodal: Whereas the large majority of participants responds more or less randomly, a small number of participants responds to a large degree with congruent choices and thereby drives the average effect (see also Eichfelder et al., 2023; Janczyk et al., 2024; Sun et al., 2020). This was taken to question the general suitability of free-choice test phases, as they may be subject to participants’ deliberations to a large degree (Custers, 2023; Custers, 2024; Janczyk et al., 2024 Kunde & Janczyk, 2024). While strategy driven response patterns still can be interpreted as evidence for learned relations between action and effect, these would be rather explicit and would not serve to provide insights into the automatically driven processes assumed in the ideomotor principle. Instead, congruency effects on forced-choice test phase RTs might be better suited to assess A-E learning in the sense of the ideomotor principle and to reveal potential generalization effects of such.

**Fig. 1:**
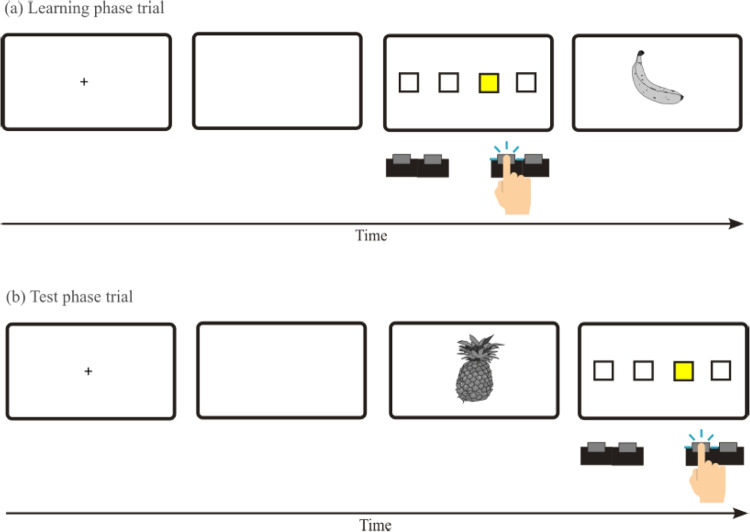
Trial structure of the original experiment of Esser et al. (2023) which is adapted in our Experiment 3. This figure illustrates the trial structure of learning phase and test phase trials in the original experiments of Esser et al. (2023), which is also used in our Experiment 3. In this example the middle-right key is mapped to the banana stimulus

Indeed, a recent study using RTs as the main dependent variable reported evidence for generalization of A-E learning to similar stimuli. In Experiment 1 of Esser et al. (2023), participants completed a forced-choice learning phase to learn specific A–E associations, followed by a forced-choice test phase to assess the presence of a congruency effect indicating A-E learning and its generalization to similar stimuli. More precisely, four horizontally aligned rectangles were displayed on the screen and served as imperative stimuli during the learning phase. On each trial, one of the rectangles was highlighted in yellow and participants were instructed to respond to its position by pressing a spatially corresponding key on a keyboard. After each response, one of four effect stimuli was shown (i.e., an image of a banana, a violin, a pig, or a dress), depending on the imperative stimulus. For example, a banana was shown whenever the left-most rectangle was highlighted (independent of the actual response). In the test phase, the order of events was reversed: Participants were first shown an image as a prime stimulus, followed by the imperative stimulus in form of the yellow-highlighted box to which participants were required to press the spatially corresponding key. Importantly, two factors were manipulated: First, the prime stimuli presented in the test phase belonged to one of three categories: (a) the original (learned) effect stimuli, (b) four new, but similar stimuli (pineapple, piano, donkey, shirt), or (c) four new, dissimilar stimuli (foot, hand, nose, ear). Second, in each trial, the prime could either be congruent with the required response or not. A congruent trial was realized when the prime was the same or a similar stimulus as that associated with the expected response in the learning phase (e.g., when the pineapple was presented ahead of the stimulus associated with the banana; for dissimilar stimuli, this assignment was arbitrary). The trial structure of this experiment was identical to our Experiment 3 and can be seen in Fig. 1.

If A-E associations were learned, shorter RTs were expected in congruent than in incongruent trials when the original, learned effects were used as primes. If these associations generalize, a comparable effect should also be obtained when the prime stimulus was similar to the effect stimulus encountered during learning, while no RT differences should result with new, dissimilar stimuli. This pattern was reported by Esser et al. (2023), that is, they observed a significant congruency effect for previously encountered (learned) and similar stimuli, but not for dissimilar ones. Experiment 2 followed the same design and analysis as Experiment 1, but used different pictures. In this case, the new similar stimuli were similar in shape rather than in category, but the results basically replicated those of Experiment 1.

As already acknowledged by Esser et al. (2023), interpretation of their results may be objected, because their learning phase trials involved two sequentially presented stimuli (the highlighted box and the effect stimulus). Thus, it is possible that the observed congruency effects resulted from learning stimulus–stimulus associations rather than genuine A–E associations. Further, a possible disadvantage of their design was that the effect depended perfectly on the stimulus rather than on the actual response. In other words, the “effect” shown on a given trial was determined by the location of the highlighted rectangle, even in trials where the participant responded incorrectly.

## The present study

Given the mixed state of results and the recent criticism of free-choice test phases (Custers, 2023; Janczyk et al., 2024; Kunde & Janczyk, 2024), the aim of the present study was to provide further empirical data on generalization of A-E learning when being assessed with forced-choice test phases and RTs as the dependent variable. More precisely, we attempted to replicate the results reported by Esser et al. (2023) while modifying the task in a way that avoids confounding stimulus–stimulus and A-E learning as a possible explanation for the observed congruency effects (and thus also the generalization effects). To this end, we report three experiments, two conceptual replications (Experiment 1 and 2) and one direct replication (Experiment 3) of Experiment 1 of Esser et al. (2023). In more detail, our Experiment 1 replaced the forced-choice learning phase with a free-choice version to eliminate the possibility of stimulus–stimulus learning. Experiment 2 reintroduced the learning phase effects during each test-phase trial in order to strengthen a possible A-E association (see Elsner & Hommel, 2001). Experiment 3 was a close replication of the original Experiment 1 of Esser et al. (2023) with only minor procedural adjustments.

## Experiment 1

Participants were instructed to randomly respond with left or right key presses in a free-choice learning phase. We reduced the number of responses relative to Esser et al. (2023), because there is evidence that using less than four responses (or effects) facilitates A-E learning (see Watson et al., 2015). Depending on the response, either the image of a banana or of a pig was presented. In the test phase, participants were first shown one of four pictures as primes: a banana or a pig (learned), or a pineapple or a donkey (new similar). Then, two rectangles appeared on the left and right side of the screen, one of them highlighted in green. Participants were to respond as quickly as possible by pressing the spatially corresponding response key. We did not use new, dissimilar stimuli in the test phase, as our focus was on examining the congruency effect and possible generalization to new, similar stimuli.

To evaluate whether A-E learning occurred, the congruency effect was tested separately for learned and similar stimuli. For the learned stimuli, we expected shorter RTs, when the required response in the test phase matched the response that had previously produced the current prime in the learning phase. Moreover, if generalization occurred, the same pattern would be expected if the new, similar prime is from the same category as the effect that was produced by the respective response before.

### Methods

#### Open practices statement

The pre-registration of Experiment 1 is available at https://aspredicted.org/jxw7-53ct.pdf and the data and analysis scripts are provided at https://osf.io/bh8ky. All statistical analyses were conducted using the R software (R Core Team, 2024, Version 4.4.1). The dplyr R package was used for data processing (Wickham et al., 2023, Version 1.1.4), alongside the purrr package (Wickham & Henry, 2023, Version 1.0.2) and the tidyr package (Wickham et al., 2024, Version 1.3.1). Bayes factors (*BF*s) were calculated using the R package BayesFactor and the default settings of the function ttestBF() (Morey & Rouder, 2024, Version 0.9.12.4.7). ANOVAs were calculated with the ezANOVA function from the ez package (Lawrence, 2016, Version 4.4.0). Outputs were formatted using the schoRsch package (Pfister & Janczyk, 2024, Version 1.11), and plots were produced using ggplot2 (Wickham, 2016, Version 3.5.2). All experiments were implemented and conducted using the OpenSesame software (Mathôt et al., 2012, Version 4.0.24).

#### Sampling plan and participants

The sample size was determined by sequential sampling based on *BF*s (Schönbrodt & Wagenmakers, 2018; Schönbrodt et al., 2015) similarly to Eichfelder et al. (2023) and Janczyk et al. (2024). The minimum sample size was $N_{\min } = 30$, and the maximum was $N_{\max } = 60$. *BF*s were computed as $BF_{+0}$, which quantifies evidence for a positive effect relative to the null hypothesis. Specifically, this corresponds to testing the hypothesis $0< \delta < \infty$ against $\delta = 0$ (rather than against $\delta \le 0$). Accordingly, values of $BF_{+0}> 1$ indicate evidence in favor of a positive effect, whereas values between 0 and 1 indicate evidence in favor of the null hypothesis. The following stopping rules were preregistered, based on $BF_{+0}$.

1. To test whether A-E learning occurred (also serving as a manipulation check), the $BF_{+0}$ for comparing mean RTs of congruent versus incongruent trials for the learned stimuli was calculated. Obtaining a $BF_{+0} < \frac{1}{6}$ would indicate that no A-E learning could be shown, even in this control condition.
2. The same $BF_{+0}$ is calculated for the condition with similar stimuli. Here, a $BF_{+0}> 6$ would indicate that generalization occurred, while a $BF_{+0} < \frac{1}{6}$ would indicate that no generalization occurred.
3. A maximum number of $N = 60$ participants has been reached.

Data were obtained from a total of 36 students of the University of Bremen. Participants were excluded if they produced more than 15% errors in the test phase or if they chose one response button in over 60% of the trials in the learning phase. Both criteria would indicate poor task engagement as the task was relatively easy and participants were instructed to chose their responses randomly (i.e., approximately 50% each) in the learning phase. These criteria led to an exclusion of 5 individuals. One additional participant’s data were removed from the analysis, because of a technical issue during the experiment.

The final sample size was $N=30$ (mean age = 25.43 years; 20 females, 10 males; 27 right-handed, 2 left-handed, 1 ambidextrous). Stopping rule 2 was fulfilled with the minimal sample size, since the evidence of A-E learning in similar stimuli reached $BF_{+0} < \frac{1}{6}$ with 30 participants. All participants signed written informed consent prior to data collection, had normal or corrected-to-normal vision, and were naïve with regard to the hypotheses.

#### Stimuli and apparatus

Stimulus presentation and response collection were controlled by a standard PC connected to a 17-inch screen in a sound-attenuated and dimly lit cabin. Four pictures from the MultiPic Database (Duñabeitia et al., 2018) were used as effect (and prime) stimuli (banana, pig, pineapple, donkey), and the German word “Los!” (Engl.: “Go!”) was used as the response prompt in the free-choice learning phase. A pair of rectangles to the left and right of the screen center served as imperative stimuli in the test phase. More precisely, one of the rectangles was filled in green color while the other one was not filled. In the original experiment of Esser et al. (2023) rectangles were filled in yellow instead. We decided for green since it is generally associated with a call to action. All stimuli and effects were presented against a black background. Responses were given by pressing the “D” or “L” key on a standard German QWERTZ keyboard with the left and right index finger, respectively. Participants were given written instructions in the beginning of each experimental phase.

#### Task and procedure

**Fig. 2:**
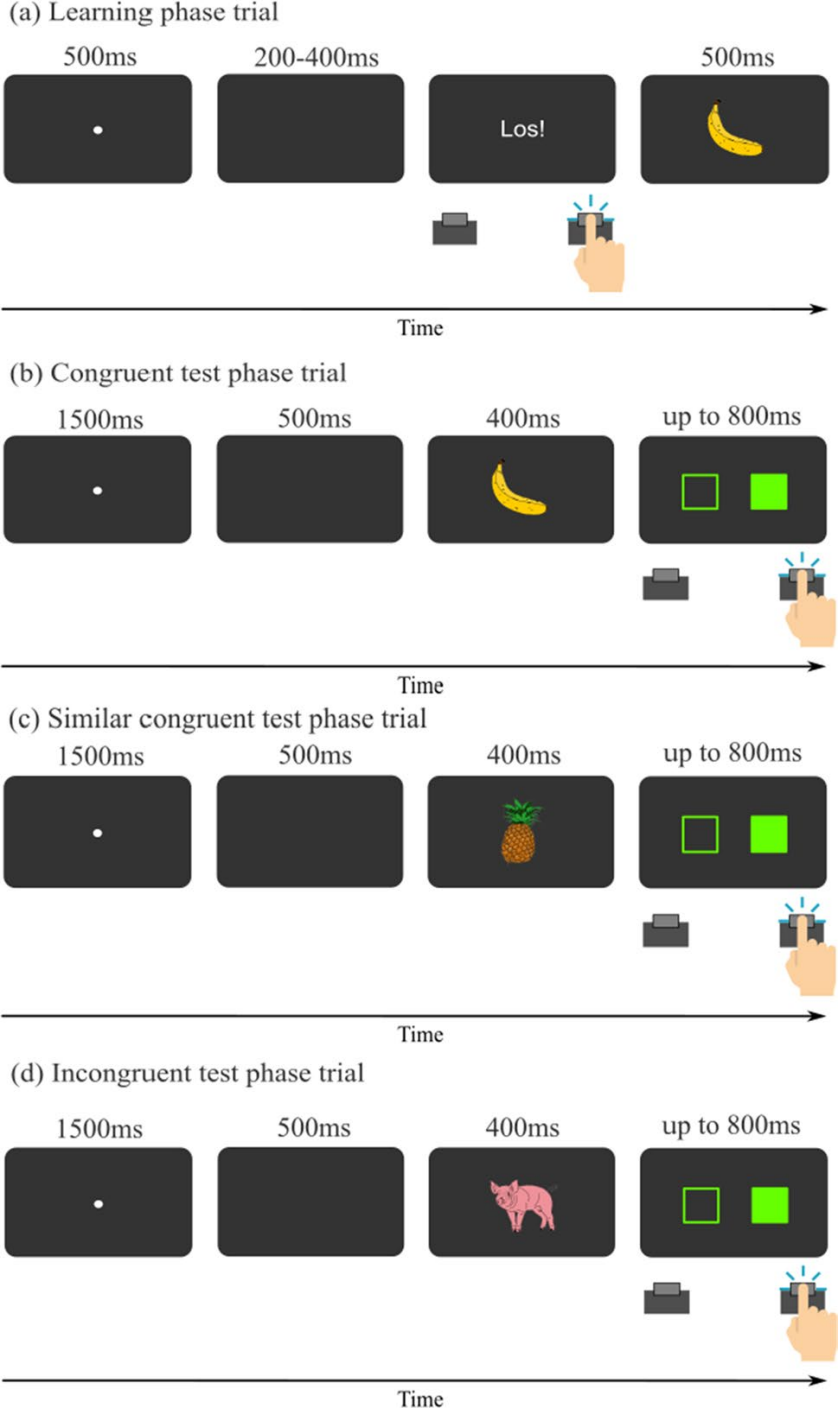
Trial structure of Experiment 1. This figure illustrates the trial structures for the learning and test phase in Experiment 1. In this example, the right key would be mapped to the stimulus “banana”

Each learning phase trial (see Fig. 2a for an illustration) began with a white fixation dot appearing in the screen center for 500ms, followed by a randomly chosen interval of 200-400ms blank screen. Afterwards, the German word “Los!” appeared in the screen center prompting the participant to respond. The response immediately triggered the central presentation of the picture of a banana or a pig for 500ms. The assignment of response key to picture was fixed for each participant, but counterbalanced across participants. If the corresponding RT was shorter than 100ms (anticipatory response), the error message “Zu schnell!” (Engl.: “too fast”) appeared on the screen. If no response was given within 1000ms (omitted response), the message “Zu langsam!” (Engl.: “too slow”) appeared instead (following previous research using free-choice phases, e.g., Eichfelder et al., 2023; Janczyk et al., 2024). Error messages were presented centrally for 500ms. The next trial started after an inter-trial interval of 1000ms. The learning phase comprised 200 trials overall.

Each test-phase trial (see Fig. 2b-d for an illustration) started with the fixation dot for 1500ms, followed by a 500ms blank screen. Then, one of the four pictures was presented for 400ms, directly followed by the pair of rectangles, which were presented for a maximum of 800ms or until a response was registered. The participants’ task was to respond with a left or right key press to the location of the green rectangle. No error feedback was provided in the test phase. All combinations of pictures and the side of the green rectangle appeared equally often in random order. The test phase also comprised 200 trials.

After finishing the experiment, participants were given a multiple choice questionnaire to determine if they can remember which key produced which effect in the learning phase. They could select whether a key produced a pig, a banana, randomly one of those images, or that the key and pictures were not associated at all. This was asked for each key individually.

#### Design and analyses

For learned stimuli, a trial was congruent when the picture preceding the imperative stimulus was the one previously produced as the effect of the required response; otherwise it was an incongruent trial. For the similar stimuli, a trial was congruent, when the picture was of the same category as the one that was previously produced by the required response. All trials following an error-trial were excluded, and for RT analyses, only correct trials were considered.

As pre-registered, Bayesian equivalents of one-tailed *t* tests were used to assess the congruency effects on RTs and PEs for the learned and the similar stimuli separately. An additional Bayesian equivalent of a one-tailed *t* test was calculated to compare the congruency effects between learned and similar stimuli. We chose a one-tailed test, since we expected positive congruency effects and a larger congruency effect for learned stimuli than for similar ones. The congruency effects were calculated as $\Delta \text {RT} = \text {RT}_\text {incongruent}-\text {RT}_\text {congruent}$ or $\Delta \text {PE} = \text {PE}_\text {incongruent}-\text {PE}_\text {congruent}$. For all analyses, the frequentist inferential statistics are reported alongside for better comparability with previous studies. However, the *BF*s are always reported first, and inferences are drawn based on the Bayesian analyses. Regarding the questionnaires, correct and wrong responses were counted and it was checked, whether the participants with wrong answers were part of the excluded participants.

### Results

#### Learning phase

Anticipatory responses occurred on average in 5.23% of a participant’s trials, and responses were omitted in 2.89%. In on average 48.7% of the remaining trials, participants responded with the left key (minimum left responses: 40.3%, maximum left responses: 55.3%, $SD = 3.66$). The mean RT for valid trials was 315ms.

#### Test phase

**Fig. 3:**
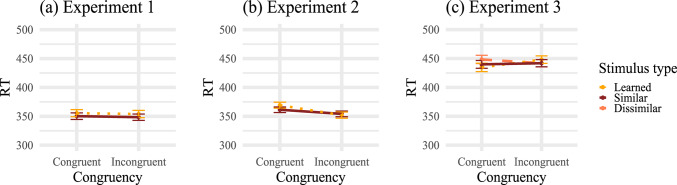
Mean (correct) response times (RTs) in milliseconds for Experiments 1-3. Mean (correct) RTs are shown as a function of congruency and stimulus type. Error bars show $\pm 1$ standard error of the mean

**Fig. 4:**
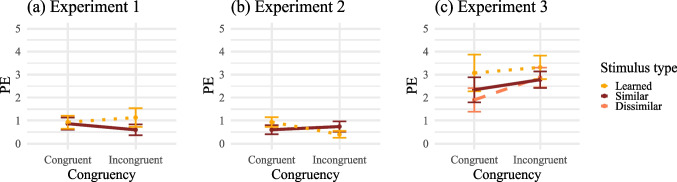
Mean percentages error (PE) for Experiments 1-3*.* PEs are shown as a function of congruency and stimulus type. Error bars show $\pm 1$ standard error of the mean

Table 1 summarizes the mean RTs and PEs for each stimulus type and congruency condition (see also Figs. 3a and 4a for a visualization). For RTs, evidence was ambiguous for a congruency effect for the learned stimuli, $BF_{+0} = 0.174$, $t(29) = -0.15$, $p =.560$, $d_z = -0.03$ and was against an effect for the similar stimuli, $BF_{+0} = 0.116$, $t(29) = -0.80$, $p =.786$, $d_z = -0.15$. Also, evidence regarding a difference in effect size between learned and similar stimuli was ambiguous, $BF_{+0} = 0.231$, $t(29) = 0.22$, $p =.585$, $d_z = 0.04$.

For PEs as well, no evidence for a congruency effect was observed regarding the learned stimuli, $BF_{+0} = 0.136$, $t(29) = -0.53$, $p =.299$, $d_z = -0.10$. For the similar stimuli, $BF_{+0} = 0.448$, $t(29) = 0.89$, $p =.810$, $d_z = 0.16$, as well as for the difference in congruency effects between conditions, $BF_{+0} = 0.553$, $t(29) = 1.07$, $p =.853$, $d_z = 0.20$, the results were ambiguous, because the observed $BF_{+0}$ values were not smaller than the threshold for evidence against an effect (i.e., $\frac{1}{6}$), but remained also below the threshold for evidence in favor of an effect (i.e., 6), as defined by the sequential sampling criteria.

**Table 1:** Mean (correct) response times (RTs) in milliseconds and percentages error (PE) for Experiment 1

	RTs			PE		
	Congruent	Incongruent	$\Delta$	Congruent	Incongruent	$\Delta$
Learned stimuli	355 (34)	354 (35)	-1	0.9 (1.6)	1.1 (2.2)	0.2
Similar stimuli	350 (31)	348 (30)	-2	0.9 (1.5)	0.6 (1.3)	-0.3

*Note.* This table shows the mean RTs and PEs for each stimulus type and congruency condition. $\Delta$ shows the difference between congruent and incongruent. The respective between subject standard deviations are written in parentheses

#### Questionnaire results

Of the 36 participants, only three failed to correctly identify which key produced which effect. One participant reported a reversed mapping between key and effect. This participant was already excluded for pressing the same key in more than 60% of all learning phase trials. In addition, one participant indicated that the effects were presented randomly after a key press, and another one reported that there was no connection between the stimulus and the effect. These two participants were not excluded from the sample, as they did not meet any of the exclusion criteria.

### Discussion

Contrary to our expectations, our data revealed neither evidence for a congruency effect with the learned stimuli nor generalization of such a congruency effect, and hence A-E learning, to similar stimuli. These results indicate that either no learning of the A-E relation occurred or that the learned associations have not been probed successfully in the test phase. In any case, Experiment 1 did not replicate the results reported by Esser et al. (2023).

## Experiment 2

Experiment 1 was not successful in replicating the congruency effect or its generalization as reported by Esser et al. (2023). One possible explanation is that the learned associations were not strong enough. Experiment 2 was therefore designed to strengthen previously acquired A-E associations. To do so, we decided to continuously reactivate A-E associations within the test phase by re-presenting the effect contingent on each response. Thus, after each response, the stimulus, which was associated with the pressed button during the learning phase, was presented again. This was also done in the “A”-experiments in the study by Elsner and Hommel (2001) and yielded larger effects in comparison to when the association was only built in the learning phase (as in their “B”-experiments). Hence, this reactivation should strengthen possible A-E associations and might thereby reveal the expected learning and generalization effects.

### Methods

#### Open practices statement

The pre-registration of Experiment 2 is available at https://aspredicted.org/5g7b-hxrb.pdf and the data and analysis scripts are provided at https://osf.io/bh8ky. Data analyses used the same software as stated for Experiment 1.

#### Sampling plan and participants

Since data collection was conducted as part of a university course, the sample size was initially fixed to 24 participants for pragmatic reasons. To allow for a better comparison with Experiment 1, we subsequently collected additional data until 30 valid data sets were gathered. To this end, data were collected from a total of 40 volunteering students of the University of Bremen. Exclusion criteria were as in Experiment 1. Overall, 10 participants were excluded from the analyses, because they used one key for more than 60% of the learning phase trials. All participants in the final sample (mean age = 25.31 years; 25 females, 5 males; 25 right-handed, 5 left-handed) had normal or corrected-to-normal vision, and were naïve with regard to the hypotheses.

#### Task, procedure, design, and analyses

The experiment was very similar to Experiment 1 and we thus only report the differences here. For the first 30 participants, stimulus presentation and response collection were controlled by a standard PC connected to a 17-inch screen in a group lab hosting up to 6 participants at the same time. Data for the remaining 10 participants were collected with a standard PC connected to a similar screen as well, but in a sound-attenuated and dimly lit cabin.

**Fig. 5:**
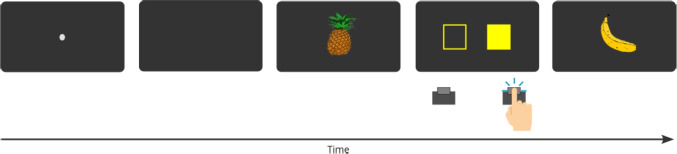
Test trial structure of Experiment 2. In this example, the right key would be mapped to the stimulus “banana”. Since the same response is expected as associated with the banana during the learning phase, it is a congruent trial

The same stimuli were used as in Experiment 1, but with yellow instead of green rectangles for more similarity to the original experiment by Esser et al. (2023). The learning phase was as in Experiment 1. In the test phase, each trial followed the procedure of Experiment 1 until the participant gave their response. Instead of directly continuing with the next trial, however, the stimulus which was associated with the given response in the learning phase was presented again for 500ms. A schematic overview of the test phase trial can be seen in Fig. 5. Trial numbers and counterbalancing were as in Experiment 1. The same analyses were conducted as in Experiment 1.

Participants completed the same questionnaire as in Experiment 1 after finishing the experiment.

### Results

#### Learning phase

Anticipatory responses occurred in on average 4.41% of a participant's trials, while responses were omitted in 3.33%. In on average 50.00% of the remaining valid trials, participants responded with the left key (minimum left responses: 42.9%, maximum left responses: 60.7%, $SD = 4.73$).^1^ The mean RT for valid trials was 326ms.

#### Test phase

Table 2 summarizes the mean RTs and PEs for each stimulus type and congruency condition (see also Figs. 3b and 4b for a visualization). As in Experiment 1, there was no evidence that RTs for congruent trials were shorter than for incongruent trials regarding learned stimuli, $BF_{+0}=0.039$, $t(29) = -6.08$, $p>.999$, $d_z = -1.11$, nor for similar stimuli, $BF_{+0} = 0.056$, $t(29) = -2.99$, $p =.997$, $d_z = -0.55$. The size of the (descriptively reversed) congruency effect was not larger for learned stimuli than for similar stimuli, $BF_{+0} = 0.052$, $t(29) = -3.33$, $p =.999$, $d_z = -0.61$.

There was no evidence that participants committed more errors in incongruent trials compared to congruent trials for learned stimuli, $BF_{+0} = 0.065$, $t(29) = -2.29$, $p =.985$, $d_z = -0.42$, nor for similar stimuli $BF_{+0} = 0.142$, $t(29) = -0.46$, $p =.674$, $d_z = -0.08$. Additionally, the (descriptive) congruency effects on PE were not larger for learned stimuli than for similar stimuli, $BF_{+0} = 0.068$, $t(29) = -2.17$, $p =.981$, $d_z = -0.40$.

**Table 2:** Mean (correct) response times (RTs) in milliseconds and percentages error (PE) for Experiment 2

	RTs			PE		
	Congruent	Incongruent	$\Delta$	Congruent	Incongruent	$\Delta$
Learned stimuli	370 (29)	353 (30)	-17	0.9 (1.2)	0.5 (0.8)	-0.4
Similar stimuli	362 (27)	353 (27)	-9	0.6 (1.1)	0.7 (1.2)	0.1

*Note.* This table shows the mean RTs and PEs for each stimulus type and congruency condition. $\Delta$ shows the difference between congruent and incongruent. The respective standard deviations are written in parentheses

#### Exploratory tests

Given the obtained results, we additionally calculated two-tailed *t* tests for all tests that resulted in $p> 0.9$ in the previous (frequentist) analyses. This was done to explore potential evidence for differences between congruent and incongruent trials in the direction contrary to our hypotheses. According to these analyses, there is very strong evidence for RTs in congruent trials to be longer than in incongruent trials for learned stimuli, $BF_{10} = 13983.620$, $t(29) = -6.08$, $p <.001$, $d_z = -1.11$, and also evidence for this effect for similar stimuli, $BF_{10} = 7.349$, $t(29) = -2.99$, $p =.006$, $d_z = -0.55$. There was strong evidence for a difference in effect sizes between learned and similar stimuli $BF_{10} = 15.408$, $t(29) = -3.33$, $p =.002$, $d_z = -0.61$ For PEs, the results were inconclusive for a difference between congruent and incongruent trials for learned stimuli, $BF_{10} = 1.841$, $t(29) = -2.29$, $p =.029$, $d_z = -0.42$ as well as for the difference between the congruency effect of learned and similar stimuli in PEs, $BF_{10} = 1.478$, $t(29) = -2.17$, $p =.038$, $d_z = -0.40$.

#### Questionnaire results

Of the 40 participants, one failed to correctly identify which key produced which effect. This participant was excluded for pressing the same key in more than 60% of the learning phase trials.

### Discussion

As in Experiment 1, RTs were not shorter in congruent than in incongruent trials, despite the additional re-presentation of effects during the test phase. In contrast, there was evidence for RTs in congruent trials to be longer than in incongruent trials for both learned and similar stimuli. In addition, there was evidence that participants made more errors in congruent trials compared to incongruent trials for learned stimuli. Together with the results obtained in Experiment 1, it appears as if no congruency effect is obtained with our design (which was based on the experiments reported by Esser et al., 2023), once learning of stimulus-stimulus associations is made impossible by using a free-choice learning phase. In contrast, the reversed congruency effect obtained in Experiment 2 suggests that an additional, unknown mechanism possibly interferes with responses in congruent trials and thereby lengthens RTs. We will come back to this in the General Discussion section.

Since both conceptual replication attempts failed to demonstrate a congruency effect even for the learned stimuli, the following Experiment 3 presents an attempt to replicate the experimental setup of Esser et al. (2023) more directly. This was done to assess whether our diverging results can be attributed to modifications introduced in Experiments 1 and 2 (most notably the use of a free-choice learning phase).

## Experiment 3

Given the results of Experiments 1 and 2, Experiment 3 aimed to replicate the Esser et al. (2023) design more directly. Hence, in the learning phase, four rectangles were now displayed on the screen, with one of them highlighted as the imperative stimulus. Participants were instructed to respond by pressing the one (out of four) spatially corresponding response key. Following the key press, one of four images (violin, banana, pig, dress) was shown, depending on the given response (instead of depending on the imperative stimulus as in the original Esser et al., 2023, study). In the test phase, a prime stimulus was presented first, followed by the four rectangles. Participants again responded to the highlighted rectangle by pressing the spatially corresponding key. The test stimuli included either one of the original learning phase effects (learned stimuli) or one of eight novel stimuli. These new stimuli were either images similar (piano, pineapple, donkey, shirt) or dissimilar (foot, hand, nose, ear) to those in the learning phase.

### Methods

#### Open practices statement

The pre-registration of Experiment 3 is available at https://aspredicted.org/xcqm-m8pb.pdf and the data and analysis scripts are provided at https://osf.io/bh8ky. We used the same software as stated for Experiment 1. Since the dissimilar stimuli are not directly related to any of the hypotheses, they were not mentioned in the pre-registration. However, we still included them in the experiment and analyses, as they were part of the original study.

#### Sampling plan and participants

Esser et al. (2023) used *t* tests to evaluate the congruency effect on test phase RTs for learned and similar stimuli with $t = 2.67$ and $t = 2.19$, respectively. Effect sizes were calculated based on $N=24$ as reported in their “Sample” section, resulting in $d_z=0.545$ for learned stimuli and $d_z=0.447$ for similar stimuli. To account for potential overestimation, we assumed an effect size of $d_z = 0.75\cdot 0.447 = 0.3352$ for a power analysis. Following these assumptions, a sample size of $N=57$ participants was required to achieve a power of $1-\beta =0.8$ with $\alpha = 0.05$ in a one-tailed *t* test. All 57 participants in the final sample (mean age = 23.89 years; 42 females, 14 males, 1 other; 51 right-handed, 5 left-handed, 1 ambidextrous) had normal or corrected-to-normal vision, and were naïve with regard to the hypotheses.

#### Task, procedure, design, and analyses

The experiment was similar to Experiments 1 and 2 with some changes applying. Twelve images from the MultiPic Database (Duñabeitia et al., 2018) were used as stimuli. The images of a violin, a banana, a pig, and a dress were used during the learning phase. During the test phase, images of a piano, a pineapple, a donkey, and a shirt were used as new similar stimuli and images of a foot, a hand, a nose, and an ear were used as dissimilar stimuli, additionally to the learning phase stimuli.

Four outlined rectangles next to each other with one rectangle being colored in yellow were used as the imperative stimuli. All stimuli and effects were shown against a white background. Responses were given by pressing the “Y”, “X”, “N” or “M” key on a standard German QWERTZ keyboard with the left and right middle and index fingers.^2^ A schematic overview of the trial structure is provided in Fig. 1. Participants completed a total of 120 learning trials and 144 test phase trials in randomized order.

Similar analyses were conducted as in the prior experiments, with additional tests for dissimilar stimuli. Moreover, a $2\times 3$ Analysis of Variance (ANOVA) with congruency (congruent vs. incongruent) and stimulus type (learned vs. similar vs. dissimilar) as repeated-measures was conducted to support the analysis, evaluate potential interactions, and to provide the same analysis as reported by Esser et al. (2023) for better comparability.

### Results

#### Learning phase

Anticipatory responses occurred in 0.1% of all trials. In none of the trials participants failed to respond in time. Since this experiment applied a forced-choice learning phase (contrary to the previous experiments), the threshold for omissions was set to 1500ms, as done in the experiment by Esser et al. (2023). The mean RT for valid trials was 434ms and wrong responses occurred in 3.6% of all trials.

#### Test phase

Table 3 summarizes the mean RTs and PEs for each stimulus type and congruency condition (see also Figs. 3c and 4c for a visualization).

**Table 3:** Mean (correct) response times (RTs) in milliseconds and percentages error (PE) for Experiment 3

	RTs			PE		
	Congruent	Incongruent	$\Delta$	Congruent	Incongruent	$\Delta$
Learned stimuli	435 (55)	448 (49)	13	3.1 (6.0)	3.3 (3.9)	0.2
Similar stimuli	440 (51)	442 (47)	2	2.3 (4.1)	2.8(2.7)	0.5
Dissimilar stimuli	448 (54)	442 (48)	-6	2.8 (3.9)	1.9 (3.2)	-0.9

*Note.* This table shows the mean RTs and PEs for each stimulus type and congruency condition. $\Delta$ shows the difference between congruent and incongruent. The respective standard deviations are written in parentheses

There was very strong evidence for a congruency effect on RTs for learned stimuli, $BF_{+0} = 162.022$, $t(56) = 3.86$, $p <.001$, $d_z =.51$, but only ambiguous evidence for similar stimuli, $BF_{+0} = 0.277$, $t(56) = 0.71$, $p =.241$, $d_z =.09$, and no evidence for dissimilar stimuli, $BF_{+0} = 0.053$, $t(56) = -1.89$, $p =.968$, $d_z = -0.25$. Additionally, there was evidence that the congruency effect was smaller for similar than for learned stimuli, $BF_{+0} = 4.271$, $t(56) = 2.44$, $p =.009$, $d_z = 0.32$, as well as strong evidence that the effect is smaller for dissimilar stimuli compared to learned stimuli, $BF_{+0} = 1896.117$, $t(56) = 4.65$, $p <.001$, $d_z = 0.62$. Evidence was ambiguous for the difference between similar and dissimilar stimuli $BF_{+0} = 1.011$, $t(56) = 1.67$, $p =.050$, $d_z = 0.22$.

Within the ANOVA, the main effect of congruency was not significant, $F(1, 56) = 3.91$, $p =.053$, $\eta _p^2 =.07$. However, the interaction between congruency and stimulus type was significant, $F(2, 112) = 9.37$, $p <.001$, $\eta _p^2 =.14$, suggesting that the effect of compatibility varied for the stimulus type conditions. The main effect of stimulus type was not significant, $F(2, 112) = 1.44$, $p =.241$, $\eta _p^2 =.03$ (sphericity was not violated for either effect).

For PEs, there was ambiguous or no evidence for a congruency effect for the learned, similar, and dissimilar stimuli with, $BF_{+0} = 0.183$, $t(56) = 0.29$, $p =.388$, $d_z =.04$, $BF_{+0} = 0.282$, $t(56) = 0.72$, $p =.236$, $d_z = 0.10$, and $BF_{+0} = 0.690$, $t(56) = 1.43$, $p =.080$, $d_z = 0.19$, respectively. The congruency effects were not larger for learned stimuli compared to similar stimuli, $BF_{+0} = 0.126$, $t(56) = -0.18$, $p =.572$, $d_z = -0.02$, nor for learned than dissimilar stimuli, $BF_{+0} = 0.093$, $t(56) = -0.65$, $p =.742$, $d_z = -0.09$, or similar than dissimilar stimuli, $BF_{+0} = 0.100$, $t(56) = -0.54$, $p =.703$, $d_z = -0.07$.

A 2 $\times$ 3 ANOVA was conducted on PEs as well. In line with previous results, there was no significant main effect of congruency, $F(1, 56) = 2.14$, $p =.149$, $\eta _p^2 =.04$, and no significant main effect of stimulus type, $F(2, 112) = 1.33$, $p =.270$, $\eta _p^2 =.02$. The interaction between compatibility and stimulus type was also not significant, $F(2, 112) = 0.25$, $p =.777$, $\eta _p^2 <.01$ (sphericity was not violated for either effect).

### Discussion

In contrast to Experiments 1 and 2, we observed a congruency effect for the learned stimuli in this experiment. Yet, no such effect was observed for similar and dissimilar stimuli. Thus, while now replicating the congruency effect for learned stimuli, we were still unable to replicate the results concerning generalization as reported by Esser et al. (2023), even though this experiment was a rather direct replication of their Experiment 1.

One procedural deviation from the original study was that, in our experiment, the effect presented during the learning phase was dependent on the participant’s actual response, rather than on the expected correct response (and hence on the stimulus). Since, this change would only impact erroneous learning phase trials (3.6% of all learning phase trials) it is unlikely the reason for the failure to replicate. Furthermore, if the congruency effect for learned stimuli and its generalization toward novel stimuli can truly be attributed to A–E learning rather than to (confounded) stimulus–stimulus learning, our modification should enhance A–E learning by ensuring consistent A–E pairings.

## General discussion

Generalization is the cognitive process that allows individuals to apply knowledge acquired in one context to new, yet similar, situations (American Psychological Association, n.d.). This phenomenon has been extensively studied across various domains in cognitive psychology. For instance, it encompasses the transfer of preferences between similar stimuli (Reichmann et al., 2023) and the attribution of effects among stimuli sharing common elements (Wheeler et al., 2006). In this present article, we focused on generalization in A–E learning, that is, whether learned knowledge from one response and its contingent effect transfers to other exemplars of the effects’ category. Evidence in favor of this has initially been reported by Hommel et al. (2003), and more recently by Esser et al. (2023) with a novel experimental design. The present study builds on this latter one. We aimed at an additional empirical contribution to assess how the results replicate when potential stimulus–stimulus learning, which was confounded with A-E learning in the original study, is ruled out.

### Summary of the main results

In Experiments 1 and 2, we implemented a free-choice learning phase to avoid stimulus-stimulus learning, that is, when the resulting effect is always contingent on the imperative stimulus (and not only on the response). In a free-choice learning phase, no other (distinct) stimulus is paired with the effect and therefore only the A-E association can be learned. In addition, we reduced the number of responses to two instead of four with the intention to facilitate A-E learning (Watson et al., 2015). In the test phase of both experiments, the prime preceding the imperative stimulus was either one of the learned effects presented in the learning phase (as a manipulation check) or a new, but similar stimulus, belonging to the same category (to assess generalization). Experiment 1 did not yield a congruency effect for the learned stimuli nor for the new similar stimuli. Experiment 2 presented the effect stimuli congruent on the response not only in the learning phase, but also throughout the test phase (see also Elsner & Hommel, 2001, for this method). The results yielded a reversed congruency effect for learned and similar stimuli, that is, RTs were even longer in congruent than in incongruent trials. Thus, in these two experiments, we did not observe evidence for A-E learning nor for its generalization, once stimulus-stimulus learning was ruled out.

Experiment 3 more directly replicated Experiment 1 of Esser et al. (2023). By using a forced-choice learning phase, we reintroduced the possibility of stimulus-stimulus learning. In addition, we now used four responses and effect stimuli, as in the original study. With these changes, we observed evidence for learning of A–E associations with the learned stimuli, but still no generalization toward similar stimuli. The additional memory questionnaires applied in Experiments 1 and 2 show that the majority of participants correctly remembered which key produced which effect. This suggests that explicitly learned relations do not necessarily lead to automatic effects, which are typically assumed in ideomotor theory. We additionally ran the analysis of Experiment 1 without all 3 participants who remembered the A-E relation incorrectly. This analyses yielded qualitatively the same results, however. In Experiment 3, no memory questionnaire was applied since we aimed to replicate the original experiment by Esser et al. (2023). However, inclusion of memory questionnaires is generally recommended for future similar studies to assess potential roles of explicit knowledge more thoroughly.

To summarize, (1) A-E learning was only visible when stimulus-stimulus learning was possible (Exp. 3) and (2) generalization was not observed in either experiment. Hence, earlier results were likely driven by stimulus–stimulus learning, not by genuine A–E association. Nonetheless, it should be noted that generalization could only truly be assessed in Experiment 3, because the underlying A–E associations were not reliably learned in Experiments 1 and 2. Therefore, the lack of generalization in these experiments does not provide strong evidence against the theoretical possibility of generalization.

To ensure that our results, in particular those relating to null effects, were not due to an insufficient ability to detect such effects, we conducted a simulation to estimate detection rates (Schönbrodt & Wagenmakers, 2018). We simulated data for the sample sizes used in Experiments 1 and 2 ($N=30$) and in Experiment 3 ($N=57$), and for multiple plausible effect sizes: a medium effect for within-subject designs (Cohen’s $d_z=0.4$), the effect size used in the sample size calculation for Experiment 3 ($d_z=0.335$), and the observed effect size in Experiment 3 ($d_z=0.51$). For each scenario, we simulated 10000 data sets and computed $BF_{+0}$, as we did in our analyses of empirical data. This allows to estimate the proportion of simulations that yielded strong evidence for a positive effect, for a null effect, or that were inconclusive. Results were considered conclusive regarding a positive effect when the Bayes factor was above the predefined thresholds. As cut-off values for evidence in favor of or against an effect, we used 6 and $\tfrac{1}{6}$, respectively, as these thresholds were applied in the main analyses of the study and the stopping rules of the sequential sampling procedure. In addition, we report results using thresholds of 3 and $\tfrac{1}{3}$, which are commonly used cut-off values in Bayesian analyses as well (Jeffreys, 1961). The results are presented in Table 4. Based on our simulations, our experimental designs show a generally low probability of yielding strong evidence against the presence of a positive effect across all examined effect sizes. Although the probabilities of obtaining strong evidence in favor of the effect are more modest, the majority of remaining outcomes fall into the ambiguous range rather than indicating evidence for the null.

Taken together, these results indicate that the evidence for the absence of an effect observed in our studies would be highly unlikely if there were indeed effects in the population. The experiments retain reasonable sensitivity to effects of the observed magnitudes. Consequently, it appears unlikely that the absence of evidence for an effect reported in the main analyses are primarily due to insufficient possibility to detect them, particularly in light of the consistent pattern of null results across conditions and experiments.

**Table 4:** Detection rates as estimated via simulation

*N*	$\delta$	BF Threshold	Evidence For	Evidence Against	Ambiguous
30	0.40	6 / 1/6	0.375	0.008	0.618
57	0.40	6 / 1/6	0.668	0.002	0.330
30	0.40	3 / 1/3	0.509	0.051	0.440
57	0.40	3 / 1/3	0.776	0.014	0.210
30	0.335	6 / 1/6	0.259	0.019	0.722
57	0.335	6 / 1/6	0.479	0.008	0.512
30	0.335	3 / 1/3	0.378	0.106	0.516
57	0.335	3 / 1/3	0.604	0.045	0.351
30	0.51	6 / 1/6	0.596	0.001	0.403
57	0.51	6 / 1/6	0.897	0.000	0.103
30	0.51	3 / 1/3	0.726	0.015	0.259
57	0.51	3 / 1/3	0.944	0.001	0.055

*Note.* Entries indicate the proportion of 10000 simulated experiments yielding evidence for, evidence against, or ambiguous evidence under according to the $BF_{+0}$ and the respective threshold

### Implications and relations with other studies

Let us first consider the results with regard to old, learned stimuli. Experiments 1 and 2 did not yield a congruency effect (we will come back to the actual reversal below), but Experiment 3 did (as did the results reported by Esser et al., 2023). One obvious difference is that the first two experiments used only 2 imperative stimuli and responses, but Experiment 3 used 4 each (again, as was the case in the study by Esser et al., 2023). Logically, then, using four stimuli and responses might be necessary to observe a congruency effect. However, such an explanation seems unlikely. First, several previous studies (Elsner & Hommel, 2001; Pfister et al., 2011; Wolfensteller & Ruge, 2011) did show congruency effects in RTs with only few possible responses and it would be indeed unclear, why a larger number of stimuli and responses should be required to do so. Second, Watson et al. (2015) showed a congruency effect in Experiment 1 with 2 A-E mappings, but not in Experiment 2 where the number of effects was changed to 4 which were either mapped to 4 or 2 response keys. This indicates that a larger amount of A-E combinations hinders A-E learning. Thus, the amount of possible A-E relations is rendered unlikely the critical difference.

The second important difference between those experiments reporting a congruency effect (our Exp. 3 and the experiments in Esser et al., 2023) and those that did not (our Exps. 1 and 2) is that the former allowed for stimulus-stimulus learning while the latter did not. In animal experiments it has been shown that associations between a stimulus and a response can be transferred to a second stimulus, if the second stimulus was previously associated with the first stimulus (usually referred to as second-order conditioning). Thus, if two stimuli are presented following each other until they become associated, and afterwards the participant is trained to respond to one of them in a certain manner, the other stimulus will activate the response as well, even though it was never directly paired with the response before (e.g., Brogden, 1939; Rizley & Rescorla, 1972). While this could potentially help explaining the diverging results, it should be noted that second-order conditioning is not found as consistently in humans (Lee, 2021). Furthermore, Moeller et al. (2019) reported no evidence for the formation of stimulus-effect associations in stimulus-response-effect episodes. However, that study investigated short term binding, where each pairing is only presented once and tested immediately after. It is not unlikely that such associations might form if pairings are repeated multiple times. Should the effects reported by Esser et al. (2023) result from stimulus-stimulus learning, this implies that their generalization effects also result from generalization of stimulus-stimulus associations rather than of A-E associations. To further investigate the role of stimulus–stimulus learning in A–E learning, it might be beneficial to include questions regarding those associations in memory questionnaires as well. This seems particularly interesting given evidence that the behavioral expression of stimulus-stimulus learning may be moderated by explicit knowledge of the underlying associations (Arunkumar et al., 2024). Even though one would not count the congruency effects as evidence for A-E learning then, one should also keep in mind that the present experiments deviate from earlier studies reporting a congruency effect attributed to A-E learning: Typically, the former effect of the acquisition phase is presented as the imperative stimulus (or at least part of it; see, e.g., Elsner & Hommel, 2004; Paelecke & Kunde, 2007; Wolfensteller & Ruge, 2011). Here, and in Esser et al. (2023), it only precedes the imperative stimulus and this might be a critical point indeed. Therefore, it seems worthwhile to explore the differences between experimental designs which directly use the previous effect as imperative stimulus and those where it is only used as a prime.

What about generalization then? If we accept stimulus-stimulus learning as the critical explanation, the generalization effects reported by Esser et al. (2023) would also be cases of generalized stimulus-stimulus learning. As a consequence, the available evidence for generalization of A-E relations would still be limited. Considering other studies, the majority of them thus would support the conclusion that generalization of A-E relations does not occur. In Experiment 1 of Janczyk et al. (2024) generalization was not observed between the words “up” and “down” and a white circle appearing at the upper or lower part of the screen. The generalization found between outlines of objects and words in Experiment 2, is likely due to phonological recording instead of generalization between words and objects. In Eichfelder et al. (2023), no generalization between a category word (“furniture”) and an exemplar word (“chair”) was observed. The only study left to report generalization in A-E learning between category and exemplar as well as similar stimuli and attributes of the stimuli is Hommel et al. (2003). The major difference between Hommel et al. (2003) and the studies mentioned above is that the similar stimuli are directly used as the imperative stimuli and are not only presented as a prime. Therefore, the stimuli gain task relevance and might receive more attention compared to a prime, which could facilitate generalization. A more general consideration is that the experimental paradigms used so far may discourage generalization. Most studies, including the present experiments, use only a small set of specific stimuli that are each paired consistently with a single effect across repeated trials (e.g. Eichfelder et al., 2023; Hommel et al., 2003). Such one-to-one pairings likely lead to specific bindings and might encourage discrimination learning rather than learning of abstract categories. Under these circumstances, participants have little reason to generalize beyond the exact stimulus–effect pairs they encounter. Generalization might be more likely if the learning phase included multiple stimuli from the same category paired with the same effect, or multiple effects from the same category paired with a single stimulus, allowing participants to gain higher-level understanding of the associations (Cohen et al., 2001; Hahn et al., 2005). Testing with novel stimuli or effects from the same category could then reveal whether such abstracted relations were learned. Designing experiments with this kind of variability might provide a better context for observing generalization in A–E learning.

### The reversed congruency effect in Experiment 2

In our Experiment 2, the congruent primes paradoxically *slowed* responses, while they should have shortened RTs as a consequence of A-E learning. Frankly, we can only speculate on why this happened. One possibility might be to assume that A-E learning was indeed successful, but with different association strengths in our three experiments. The activation of the primed response then is inhibited depending on the strength of the association, thus affecting the accessibility of congruent responses. More precisely, associations might be weak in Experiment 3 due to the 4-4 mapping (see also Watson et al., 2015), intermediate in Experiment 1 with a 2-2 mapping, and strong in Experiment 2 with the re-presentation of the effect during the test phase. If the induced response becomes always inhibited some time after the prime presentation, this inhibition might be strongest in Experiment 2, lengthening congruent RTs to such a degree that the congruency effect reverses. Inhibition would be weakest in Experiment 3, without much affecting the response’s activation and thus yielding the standard congruency effect. In Experiment 1, finally, this inhibition is intermediate and reduces the response’s accessibility just to a degree that no congruency effect remains measurable in RTs. As stated, this is a very tentative post-hoc interpretation that warrants further research. At the very least some form of replication of the unexpected reversed congruency is needed to support our speculations.

### Conclusion

Three experiments were reported in an attempt to replicate generalization of A-E learning as reported by Esser et al. (2023). Overall, the results indicate that stimulus-stimulus instead of A-E learning was a critical factor in their study. In addition, we did not obtain evidence for generalization of A-E learning. However, generalization could only be meaningfully assessed in Experiment 3, since no reliable A–E associations were revealed in Experiments 1 and 2. Therefore, while we did not observe generalization, the overall evidence regarding generalization of A–E learning remains unclear as well.

## Data Availability

Data of all three experiments can be found at https://osf.io/bh8ky.

## Footnotes

1 In all experiments, participants were excluded based on response bias prior to removing error trials. Consequently, one participant who fell below the 60% cutoff in the raw data marginally exceeded it in the final sample after error trials were removed.

2 The original study by Esser et al. (2023) used “Z” instead of “Y” since they used a QWERTY keyboard. The relative placement of the key on the keyboard is identical, however.

## References

[CR1] American Psychological Association. (n.d.). Cognitive generalization. Retrieved from https://dictionary.apa.org/cognitive-generalization

[CR2] Arunkumar, M., Rothermund, K., & Giesen, C. G. (2024). One link to link them all: Indirect response activation through stimulus–stimulus associations in contingency learning. *Experimental Psychology,**70*(5), 259–270. 10.1027/1618-3169/a000597 38288913 10.1027/1618-3169/a000597 PMC10918695

[CR3] Badets, A., Koch, I., & Phillip, A. (2016). A review of ideomotor approaches to perception, cognition, action, and language: Advancing a cultural recycling hypothesis. *Psychological Research,**80*(1), 1–15. 10.1007/s00426-014-0643-8 25535019 10.1007/s00426-014-0643-8

[CR4] Berlyne, D. (1957). Conflict and choice time. *British Journal of Psychology,**48*(2), 106–118. 10.1111/j.2044-8295.1957.tb00606.x 13426496 10.1111/j.2044-8295.1957.tb00606.x

[CR5] Brogden, W. J. (1939). Sensory pre-conditioning. *Journal of Experimental Psychology,**25*(4), 323–332. 10.1037/h0058944

[CR6] Carpenter, W. (1852). On the influence of suggestion in modifying and directing muscular movement, independently of volition. *Royal Institution of Great Britain (Weekly Evening Meeting)*, 147–154.

[CR7] Cohen, A. L., Nosofsky, R. M., & Zaki, S. R. (2001). Category variability, exemplar similarity, and perceptual classification. *Memory & Cognition,**29*(8), 1165–1175. 10.3758/BF03206386 11913753 10.3758/bf03206386

[CR8] Custers, R. (2023). Thoughts about actions and outcomes (and what they lead to). *Motivation Science,**9*(4), 261–271. 10.1037/mot0000306

[CR9] Custers, R. (2024). The homunculus in the room: A reply to Kunde and Janczyk (2024) and Hommel and Eder (2024). *Motivation Science,**10*(4), 403–406. 10.1037/mot0000377

[CR10] Duñabeitia, J. A., Crepaldi, D., Meyer, A. S., New, B., Pliatsikas, C., Smolka, E., & Brysbaert, M. (2018). Multipic: A standardized set of 750 drawings with norms for six european languages. *Quarterly Journal of Experimental Psychology*, *71*(4), 808–816. (Original work published 2006). 10.1080/17470218.2017.1310261. 10.1080/17470218.2017.1310261 28326995

[CR11] Eichfelder, L., Franz, V. H., & Janczyk, M. (2023). Is there hierarchical generalization in response-effect learning? *Experimental Brain Research,**241*(1), 135–144. 10.1007/s00221-022-06473-w 36394593 10.1007/s00221-022-06473-w PMC9870827

[CR12] Elsner, B., & Hommel, B. (2001). Effect anticipation and action control. *Journal of Experimental Psychology: Human Perception and Performance,**27*(1), 229–240. 10.1037/0096-1523.27.1.229 11248937 10.1037//0096-1523.27.1.229

[CR13] Elsner, B., & Hommel, B. (2004). Contiguity and contingency in action-effect learning. *Psychological Research,**68*(2), 138–154. 10.1007/s00426-003-0151-8 14685854 10.1007/s00426-003-0151-8

[CR14] Esser, S., Haider, H., Lustig, C., Tanaka, T., & Tanaka, K. (2023). Action–effect knowledge transfers to similar effect stimuli. *Psychological Research,**87*(7), 2249–2258. 10.1007/s00426-023-01800-4 36821009 10.1007/s00426-023-01800-4 PMC10457235

[CR15] Greenwald, A. (1970). Sensory feedback mechanisms in performance control: With special reference to the ideo-motor mechanism. *Psychological Review,**77*(2), 73–99. 10.1037/h0028689 5454129 10.1037/h0028689

[CR16] Hahn, U., Bailey, T. M., & Elvin, L. B. C. (2005). Effects of category diversity on learning, memory, and generalization. *Memory & Cognition,**33*(2), 289–302. 10.3758/BF03195318 16028584 10.3758/bf03195318

[CR17] Harleß, E. (1861). Der Apparat des Willens [the apparatus of will]. *Zeitschrift für Philosophie und Philosophische Kritik,**38*, 50–73.

[CR18] Herbart, J. (1825). *Psychologie als Wissenschaft neu gegründet auf Erfahrung, Metaphysik und Mathematik [Psychology as a science newly founded on experience, metaphysics, and mathematics]*. August Wilhelm Unzer.

[CR19] Hommel, B., Alonso, D., & Fuentes, L. (2003). Acquisition and generalization of action effects. *Visual Cognition,**10*(8), 965–986. 10.1080/13506280344000176

[CR20] Hommel, B., Müsseler, J., Aschersleben, G., & Prinz, W. (2001). The theory of event coding (tec): A framework for perception and action planning. *Behavioral and Brain Sciences,**24*(5), 849–878. 10.1017/S0140525X01000103 12239891 10.1017/s0140525x01000103

[CR21] James, W. (1890). *The principles of psychology*. Harvard University Press. 10.1037/10538-000

[CR22] Janczyk, M., Eichfelder, L., Liesefeld, H. R., & Franz, V. H. (2024). Learning and transfer of response–effect relations. *Quarterly Journal of Experimental Psychology*. 10.1177/17470218241284259 10.1177/17470218241284259 PMC13168614 39256971

[CR23] Janczyk, M., Giesen, C. G., Moeller, B., Dignath, D., & Pfister, R. (2023). Perception and action as viewed from the theory of event coding: A multi-lab replication and effect size estimation of common experimental designs. *Psychological Research,**87*(4), 1012–1042. 10.1007/s00426-022-01705-8 35978172 10.1007/s00426-022-01705-8 PMC9385094

[CR24] Jeffreys, H. (1961). *Theory of probability* (3rd ed.). Oxford: Oxford University Press.

[CR25] Kunde, W., & Janczyk, M. (2024). Thoughts about “Thoughts about actions and outcomes": Comment on Custers (2023). *Motivation Science,**10*(4), 392–397. 10.1037/mot0000341

[CR26] Lawrence, M. A. (2016). Ez: Easy analysis and visualization of factorial experiments. R package version 4.4-0. Retrieved from https://CRAN.R-project.org/package=ez.

[CR27] Lee, J. C. (2021). Second-order conditioning in humans. *Frontiers in Behavioral Neuroscience,**15*, 672628. 10.3389/fnbeh.2021.672628 34305546 10.3389/fnbeh.2021.672628 PMC8295922

[CR28] Mathôt, S., Schreij, D., & Theeuwes, J. (2012). Opensesame: An open-source, graphical experiment builder for the social sciences. *Behavior Research Methods,**44*(2), 314–324. 10.3758/s13428-011-0168-7 22083660 10.3758/s13428-011-0168-7 PMC3356517

[CR29] Moeller, B., Pfister, R., Kunde, W., & Frings, C. (2019). Selective binding of stimulus, response, and effect features. *Psychonomic Bulletin & Review,**26*(5), 1627–1632. 10.3758/s13423-019-01646-1 31325038 10.3758/s13423-019-01646-1

[CR30] Morey, R. D., & Rouder, J. N. (2024). Bayesfactor: Computation of bayes factors for common designs. R package version 0.9.12-4.7. Retrieved from https://CRAN.R-project.org/package=BayesFactor.

[CR31] Paelecke, M., & Kunde, W. (2007). Action-effect codes in and before the central bottleneck: Evidence from the psychological refractory period paradigm. *Journal of Experimental Psychology: Human Perception and Performance,**33*(3), 627–644. 10.1037/0096-1523.33.3.627 17563226 10.1037/0096-1523.33.3.627

[CR32] Pfister, R., & Janczyk, M. (2024). Schorsch: Tools for analyzing factorial experiments. R package version 1.11. Retrieved from https://CRAN.R-project.org/package=schoRsch.

[CR33] Pfister, R., Kiesel, A., & Hoffmann, J. (2011). Learning at any rate: Action–effect learning for stimulus-based actions. *Psychological Research,**75*(1), 61–65. 10.1007/s00426-010-0288-1 20490862 10.1007/s00426-010-0288-1

[CR34] R Core Team. (2024). R: A language and environment for statistical computing. R Foundation for Statistical Computing. Vienna, Austria. Retrieved from https://www.R-project.org/.

[CR35] Reichmann, K., Hütter, M., Kaup, B., & Ramscar, M. (2023). Variability and abstraction in evaluative conditioning: Consequences for the generalization of likes and dislikes. *Journal of Experimental Social Psychology,**108*, 104478. 10.1016/j.jesp.2023.104478

[CR36] Rizley, R. C., & Rescorla, R. A. (1972). Associations in second-order conditioning and sensory preconditioning. *Journal of Comparative and Physiological Psychology,**81*(1), 1–11. 10.1037/h0033333 4672573 10.1037/h0033333

[CR37] Schönbrodt, F. D., & Wagenmakers, E.-J. (2018). Bayes factor design analysis: Planning for compelling evidence. *Psychonomic Bulletin & Review,**25*(1), 128–142. 10.3758/s13423-017-1230-y 28251595 10.3758/s13423-017-1230-y

[CR38] Schönbrodt, F. D., Wagenmakers, E.-J., Zehetleitner, M., & Perugini, M. (2015). Sequential hypothesis testing with bayes factors: Efficiently testing mean differences. *Psychological Methods,**22*(2), 322–339. 26651986 10.1037/met0000061

[CR39] Shin, Y. K., Proctor, R., & Capaldi, E. (2010). A review of contemporary ideomotor theory. *Psychological Bulletin,**136*(6), 943–974. 10.1037/a0020541 20822210 10.1037/a0020541

[CR40] Sun, D., Custers, R., Marien, H., & Aarts, H. (2020). Ideomotor action: Evidence for automaticity in learning, but not execution. *Frontiers in Psychology,**11*, 185. 10.3389/fpsyg.2020.00185 32116968 10.3389/fpsyg.2020.00185 PMC7033682

[CR41] Watson, P., Van Steenbergen, H., De Wit, S., Wiers, R. W., & Hommel, B. (2015). Limits of ideomotor action–outcome acquisition. *Brain Research,**1626*, 45–53. 10.1016/j.brainres.2015.02.020 25704203 10.1016/j.brainres.2015.02.020

[CR42] Wheeler, D. S., Amundson, J. C., & Miller, R. R. (2006). Generalization decrement in human contingency learning. *Quarterly Journal of Experimental Psychology,**59*(7), 1212–1223. 10.1080/17470210600576342 10.1080/17470210600576342 16769621

[CR43] Wickham, H. (2016). Ggplot2: Elegant graphics for data analysis. Springer-Verlag, New York. Retrieved from https://ggplot2.tidyverse.org.

[CR44] Wickham, H., François, R., Henry, L., Müller, K., & Vaughan, D. (2023). Dplyr: A grammar of data manipulation. R package version 1.1.4. Retrieved from https://CRAN.R-project.org/package=dplyr.

[CR45] Wickham, H., & Henry, L. (2023). Purrr: Functional programming tools. R package version 1.0.2. Retrieved from https://CRAN.R-project.org/package=purrr.

[CR46] Wickham, H., Vaughan, D., & Girlich, M. (2024). Tidyr: Tidy messy data. R package version 1.3.1. Retrieved from https://CRAN.R-project.org/package=tidyr.

[CR47] Wolfensteller, U., & Ruge, H. (2011). On the timescale of stimulus-based action–effect learning. *Quarterly Journal of Experimental Psychology,**64*(7), 1273–1289. 10.1080/17470218.2010.546417 10.1080/17470218.2010.546417 21416458

